# Genome-wide miRNA profiling and pivotal roles of miRs 125a-5p and 17-92 cluster in human neutrophil maturation and differentiation of acute myeloid leukemia cells

**DOI:** 10.18632/oncotarget.27123

**Published:** 2019-09-03

**Authors:** El-Habib Dakir, Faustino Mollinedo

**Affiliations:** ^1^Instituto de Biología Molecular y Celular del Cáncer, Centro de Investigación del Cáncer, Consejo Superior de Investigaciones Científicas (CSIC)-Universidad de Salamanca, Salamanca, Spain; ^2^Faculty of Biology, University of Latvia, Riga, Latvia; ^3^Laboratory of Cell Death and Cancer Therapy, Department of Molecular Biomedicine, Centro de Investigaciones Biológicas, CSIC, Madrid, Spain

**Keywords:** miRNA, miR-125a-5p, miR-17-92, neutrophil differentiation, human

## Abstract

MicroRNAs (miRNAs, miRs) are short non-coding post-transcriptional regulators of gene expression in normal physiology and disease. Acute myeloid leukemia is characterized by accumulation of malignantly transformed immature myeloid precursors, and differentiation therapy, used to overcome this differentiation blockage, has become a successful therapeutic option. The human HL-60 acute leukemia cell line serves as a cell culture model for granulocytic maturation, and dimethyl sulfoxide (DMSO) incubation leads to its differentiation towards neutrophil-like cells, as assessed by biochemical, functional and morphological parameters. DMSO-induced HL-60 cell differentiation constitutes an excellent model to examine molecular processes that turn a proliferating immortal leukemic cell line into mature non-proliferating and apoptosis-prone neutrophil-like end cells. By performing genome-wide miRNA profiling and functional assays, we have identified a signature of 86 differentially expressed canonical miRNAs (51 upregulated; 35 downregulated) during DMSO-induced granulocytic differentiation of HL-60 cells. Quantitative real-time PCR was used to validate miRNA expression. Among these differentially expressed canonical miRNAs, we found miR-125a-5p upregulation and miR-17-92 cluster downregulation acted as major regulators of granulocytic differentiation in HL-60 cells. Enforced expression of miR-125a-5p promoted granulocytic differentiation in HL-60 cells, whereas miR-17-92 ectopic expression inhibited DMSO-induced HL-60 granulocytic differentiation. Ectopic expression of miR-125a-5p also promoted granulocytic differentiation in human acute promyelocytic leukemia NB4 cells, as well as in naïve human primary CD34^+^-hematopoietic progenitor/stem cells. These findings provide novel molecular insights into the identification of miRNAs regulating granulocytic differentiation of human leukemia cells and normal CD34^+^-hematopoietic progenitor/stem cells, and may assist in the development of novel miRNA-targeted therapies for leukemia.

## INTRODUCTION

Polymorphonuclear neutrophils (PMNs) play an essential role in acute inflammation, are the primary mediators of the innate immune response to invading microorganisms, providing the first-line defense against infection, and have been implicated in the pathogenesis of a number of human diseases [1–4]. PMNs are the most abundant circulating blood leukocytes in adult peripheral blood, accounting for 50-70% of the white blood cell differential count [4]. Mature neutrophils enter the bloodstream and tissues as terminally differentiated non-proliferating cells, which have the shortest lifespan of all circulating leukocytes, previously estimated as about 1 day [5, 6], and recently challenged to be 5.4 days [7], as a result of constitutive and spontaneous apoptosis [8]. These proapoptotic mature neutrophils are constantly renewed through a sustained generation of neutrophils by the bone marrow at impressive numbers (10^11^ cells per day in a normal adult) through a highly controlled, yet incompletely understood process of granulopoiesis [9].

MicroRNAs (miRNAs, miRs) are endogenous small noncoding RNAs of ~18-25 nucleotides that regulate gene expression negatively by binding preferentially to the 3’-untranslated regions of their target mRNAs, leading to mRNA degradation or translational repression, and acting in this way as major post-transcriptional regulators of genes involved in fundamental processes, including apoptosis, proliferation and differentiation [10]. Several miRNAs have been involved in the differentiation and function of various hematopoietic lineages, including miR-150, miR-155 and miR-181 in the lymphoid lineage [11], and miR-223 in the myeloid lineage, particularly in granulocytes [12, 13]. Accumulating evidence has revealed that some cancers have been associated with altered expression of particular miRNAs, and some miRNA signatures have been linked with diagnosis, staging, progression, prognosis and response to treatment in certain tumors [14, 15].

Despite recent evidence has shown the importance of miRNAs in immune cell function and development, there is a shortage of information regarding how miRNAs regulate neutrophil development. A few miRNAs have been suggested to be involved in granulopoiesis, but no conclusive evidence has been attained so far [16–18].

A number of human myeloid leukemia cell lines, such as HL-60, NB4, PL21 and PLB-985, have been used to study neutrophil biology [19–22]. The human acute myeloid leukemia (AML) HL-60 cell line, derived from a patient considered initially to have acute promyelocytic leukemia [23], but later on reclassified as acute myeloblastic leukemia with maturation [24], has been widely used as a cell culture model for studies on neutrophil differentiation and function [21, 25, 26]. HL-60 cells can be differentiated towards cells sharing several functional features of mature neutrophils, which then eventually die through spontaneous apoptosis [8, 21, 25, 27]. Induction of HL-60 cell differentiation towards neutrophilic lineage can be attained following incubation with different agents, including dimethyl sulfoxide (DMSO) [21, 25]. Incubation of HL-60 cells with DMSO for 24 h is sufficient to commit cells towards granulocytic differentiation [28], and cells acquire most of the neutrophil functional and biochemical features, including their capacity to undergo spontaneous apoptosis, after a 4-day DMSO incubation [8, 21]. Previous microarray RNA analyses have suggested that neutrophil differentiation takes place through coordinated, complex and successive changes in gene expression that eventually lead to the acquisition of a mature neutrophil phenotype prone to undergo apoptosis [29].

Here, following the analysis of the role of miRNAs during the differentiation of HL-60 cells through the neutrophilic lineage, we have found a novel miRNA signature during neutrophil differentiation, being upregulation of miR-125a-5p critical for the differentiation of myeloid leukemia cell lines (HL-60 and NB4) as well as of primary CD34^+^-hematopoietic progenitor/stem cells (CD34^+^-HPCs) into human neutrophil-like cells. Furthermore, downregulation of the miR-17-92 cluster was also required for the granulocytic differentiation of HL-60 cells.

## RESULTS

### DMSO-induced granulocytic differentiation of HL-60 cells

**Figure 1:**
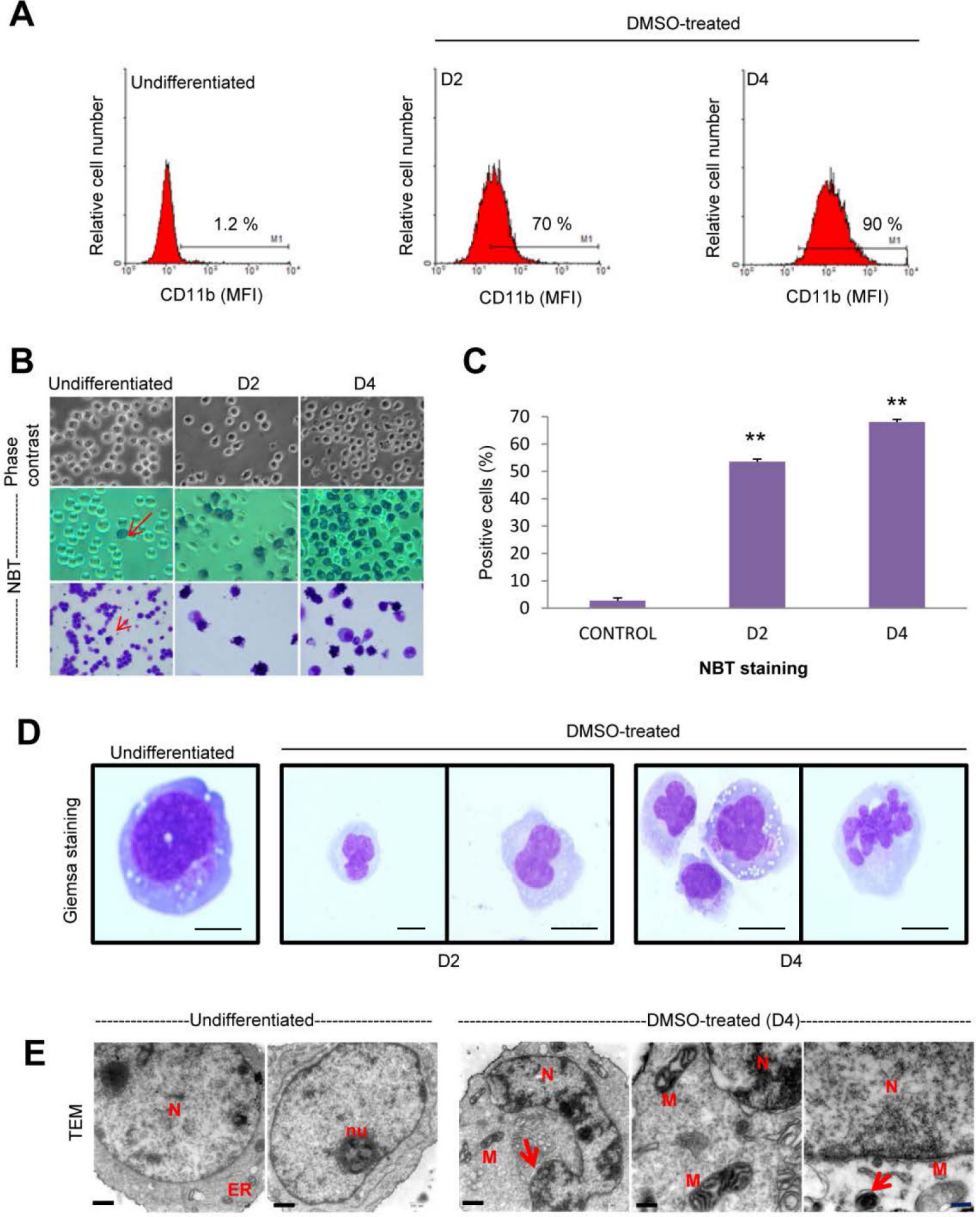
Morphological and functional changes in DMSO-induced HL-60 differentiation towards the granulocytic lineage. **(A)** Induction of HL-60 differentiation towards neutrophil lineage following incubation with 1.3% (v/v) DMSO for 2 (D2) and 4 (D4) days, as assessed by the expression of maturation-associated myeloid cell surface marker CD11b through flow cytometry analysis, measuring mean fluorescence intensity (MFI) of anti-CD11b antibody staining. Untreated control samples (undifferentiated) were run in parallel. Data shown are representative of three independent experiments. **(B)** Representative light microscopy photographs (400x magnification) of HL-60 cells untreated (undifferentiated) and DMSO-treated for 2 (D2) and 4 (D4) days, showing changes in cell morphology (upper, phase contrast) and an increase in nitroblue tetrazolium (NBT) staining during neutrophil differentiation (middle, smears; lower, cytospins). Undifferentiated cells show very few NBT-stained cells (arrow) and the percentage of NBT-positive cells, showing NBT reduction and the corresponding intracellular dark blue formazan deposits, increases along the differentiation process towards the granulocytic lineage. Data shown are representative of three independent experiments. **(C)** Quantitative measurements of the percentages of NBT-positive cells in untreated control cells and during DMSO-induced granulocytic differentiation for 2 (D2) and 4 (D4) days. Data shown are means ± SD of three independent experiments. ^**^, *p* <0.01, with respect to untreated control cells. **(D)** Cell morphology and nuclear shape of undifferentiated and DMSO-treated HL-60 cells (D2 and D4), as assessed by Wright-Giemsa staining of cytospins. *Bars*, 5 μm. **(E)** Transmission electron microscopy of HL-60 cells untreated (undifferentiated) and treated with DMSO for 4 days (D4). Undifferentiated cells show a large nucleus (*N*) (round to oval shape) with diffuse chromatin, a prominent nucleolus (*nu*) and visible rough endoplasmic reticulum (*ER*). In DMSO-treated cells, the nucleus (*N*) shows ultrastructural changes with multilobed or folded nucleus with pyknotic nuclear chromatin, and some large mitochondria (*M*) displaying electron-dense and multiple cristae. Autophagosome-like structures (arrow) are also observed. *Bar*, 500 nm.

Treatment of human leukemic HL-60 cells with DMSO (1.3% v/v) for 2 and 4 days induced granulocytic differentiation as evidenced by an increase in the cell surface expression of CD11b (Figure 1A) as well as by morphological and functional changes. Phase-contrast light microscopy showed that cells acquired the ability to generate superoxide anion during DMSO-induced neutrophil-like differentiation, as assessed by NBT staining (Figure 1B and 1C). These data are in agreement with previous reports showing neutrophil-like differentiation of HL-60 cells by DMSO treatment [8, 21, 29]. The nucleus turned into a distinguishable polymorphic, multi-lobed and segmented one, a typical hallmark for neutrophils, along the differentiation process (Figure 1D). In addition, electron microscopy analysis, during DMSO-induced granulocytic differentiation of HL-60 cells, showed the appearance of nuclei with a higher condensed chromatin, electron-dense mitochondria and autophagosome-like vacuoles with double-membrane structure during the neutrophil-like differentiation process (Figure 1E), which could be in agreement with recent reports suggesting the involvement of autophagy during neutrophil development [30–34]. Differentiated HL-60 cells following a 4-day incubation showed no, or very little, apoptosis (1.5%), but this percentage of apoptotic cells was increased to about 25% following a 5-day incubation with DMSO (Supplementary Figure 1), in agreement with previous reports [21, 29], thus indicating that DMSO-differentiated behaved as end cells prone to undergo apoptosis, like their physiological mature neutrophil counterparts, and 4-day DMSO treatment was the threshold for the acquisition of the apoptosis-prone phenotype, characteristic of mature neutrophils.

### Differentially expressed miRNAs during DMSO-induced granulocytic differentiation of HL-60 cells

**Figure 2:**
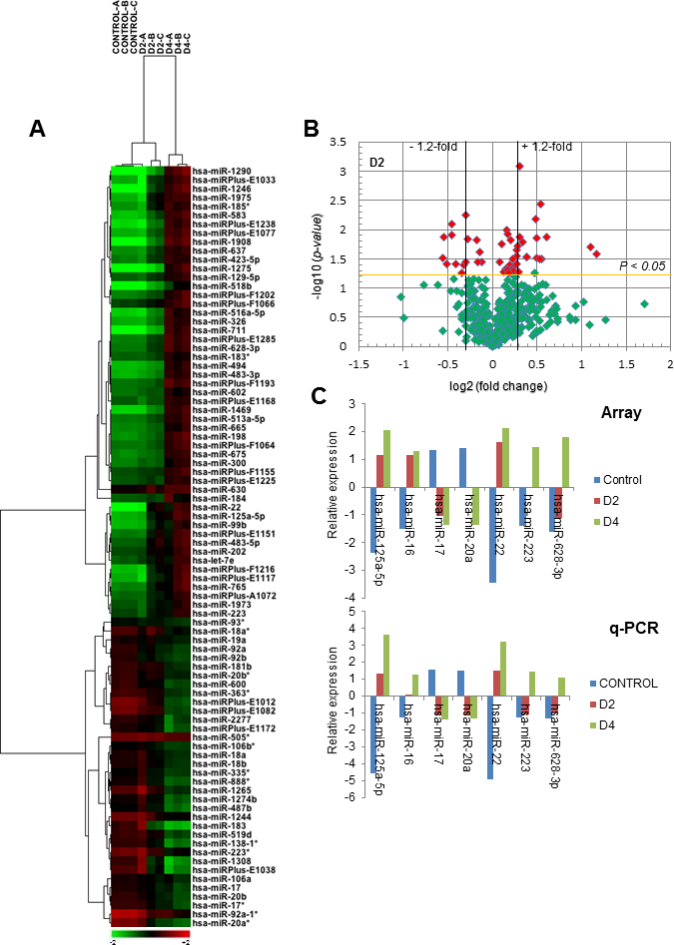
miRNA expression analysis during DMSO-induced HL-60 differentiation towards the granulocytic lineage. **(A)** Hierarchical cluster analysis of differentially expressed miRNAs in untreated control versus DMSO-treated HL-60 cells for 2 (D2) and 4 (D4) days. The heat map shows the classification of untreated and DMSO-treated cells in different subclusters. Rows: differentially expressed miRNAs; columns: untreated (Control) and DMSO-treated HL-60 cells for 2 (D2) and 4 (D4) days. Negative fold change (miRNAs with decreased expression) is represented in green and positive fold change (miRNAs with increased expression) is represented in red. Three independent experiments were performed for each differentiation stage (Control, A-C; D2, A-C; D4, A-C). MiR^*^ nomenclature (an asterisk appended to the miRNA name) indicates a mature miRNA that originates from the same hairpin of the main miRNA, is complementary to the main miRNA and is less predominant than the opposite arm (miRNA without asterisk) of the hairpin. **(B)** Volcano plot analysis of the differentially expressed miRNAs (log 2 scale) versus the corresponding *p* values (-log 10 scale), following DMSO-induced differentiation of HL-60 cells for 2 days. Red spots represent the most significantly altered miRNAs (cut-off *p* values <0.05). Horizontal and vertical lines show the thresholds used for analysis. Dots at the right upper side of the plot represent the statistically significant upregulated miRNAs, whereas those at the left upper side represent the miRNAs that were downregulated in DMSO-induced differentiated HL-60 cells for 2 days (D2). **(C)** Quantitative real-time PCR (q-PCR) validation of the array results, using seven selected miRNAs during DMSO-induced HL-60 cell differentiation. Mean values of the relative expression of each indicated miRNA from the Exiqon microarrays (Array) and quantitative PCR (q-PCR) data of untreated control and DMSO-treated HL-60 cells for 2 (D2) and 4 (D4) days are shown. Values represent averages of three independent experiments; the SD was less than 8%. Relative expression of miRNAs was calculated as described in the Materials and Methods section.

In order to investigate the role of miRNAs in the above neutrophil-like differentiation process, we analyzed the miRNA expression profile of undifferentiated and DMSO-induced differentiated HL-60 cells, using the miRCURY LNA array v.14 platform, which contains 1,280 probes complementary to mature forms of miRNAs of human origin, from highly purified high and low molecular weight RNA samples (Figure 2A). High-quality RNA preparations (Supplementary Figure 2) were used to analyze miRNA expression from untreated control HL-60 cells as well as following incubation of HL-60 cells with 1.3% DMSO for 2 days (shortly after the 1-day commitment stage required to induce differentiation towards neutrophil-like cells) [8, 21], and for 4 days (fully differentiated cells acquiring the mature and functional phenotype of neutrophil-like cells) [8, 21]. miRNAs with at least 1.2-fold changes in their expression between undifferentiated and DMSO-treated cells were considered to be differentially expressed and regulated. Among the 1,280 human miRNAs analyzed, we identified a unique signature of 86 differentially expressed miRNAs between control and DMSO-treated cells (*p* <0.05), 51 of them were upregulated and 35 were downregulated (Figure 2A and Table 1), which has not been previously identified. Hierarchical clustering analysis of the 86 differentially expressed miRNAs clearly identified three clusters, corresponding to undifferentiated control HL-60 cells, 2-day DMSO-treated HL-60 cells and 4-day DMSO-treated HL-60 cells (Figure 2A). The whole list of differentially expressed miRNAs during DMSO-induced neutrophil-like differentiation of HL-60 cells is shown in Table 1. Volcano plot analyses of the differentially expressed genes (log 2 scale) *versus* the *p*-values of the genes (-log 10 scale) were also carried out, showing highly significant differentially expressed (up and downregulated) miRNAs after 2-day (Figure 2B) and 4-day DMSO treatment (Supplementary Figure 3). Interestingly, only four miRNAs (miR-1290, miR-1246, miR-22 and miR-125a-5p) were upregulated ≥2-fold after 2-day DMSO incubation, likely at the neutrophilic commitment stage of granulocytic differentiation (Table 1). In addition, changes in all the miRNAs shown in Table 1 were either progressively increased or decreased, along the DMSO-induced differentiation process. Thus, the fold change in the upregulated miRNAs was enhanced with the incubation time of DMSO, whereas it was diminished with the DMSO incubation time in the downregulated miRNAs. This suggests that the DMSO-induced neutrophilic differentiation of HL-60 cells is a continuum process till reach mature differentiated neutrophil-like cells.

**Table 1:** Differentially expressed miRNAs during DMSO-induced differentiation of HL-60 cells

Probe ID	MicroRNA	FC (D2/C)	FC (D4/C)	Regulation
46921	hsa-miR-1290	2.122910709	18.09037363	up
46620	hsa-miR-1275	1.374211464	10.81177701	up
46514	hsa-miR-1246	2.424220302	10.48281859	up
146196	hsa-miR-711	1.560976282	8.569353624	up
28431	hsa-miR-1908	1.661720707	8.559068663	up
11020	hsa-miR-22	3.260414223	6.067323963	up
46256	hsa-miRPlus-E1238	1.442643896	5.36820652	up
146072	hsa-miR-1469	1.383513973	5.220192687	up
17295	hsa-miR-583	1.652225508	5.049910072	up
42550	hsa-miR-516a-5p	1.324161673	4.631436437	up
46580	hsa-miRPlus-E1077	1.408074008	4.560555483	up
145985	hsa-miRPlus-F1216	1.826082501	4.315373196	up
146162	hsa-miR-1975	1.842072463	4.311733226	up
**10928**	**hsa-miR-125a-5p**	**2.017336326**	**4.306794056**	**up**
46749	hsa-miRPlus-E1033	1.389686293	4.139109731	up
145901	hsa-miR-494	1.214495097	4.048893407	up
145725	hsa-miR-518b	1.415541914	4.043126509	up
146076	hsa-miR-483-3p	1.279973124	3.990345619	up
46733	hsa-miRPlus-E1117	1.516951106	3.938475948	up
42693	hsa-miR-326	1.262196726	3.81428038	up
27540	hsa-miR-198	1.290487048	3.813389107	up
11184	hsa-miR-99b	1.903775273	3.414961521	up
17904	hsa-miR-185^*^	1.5806679	3.353804851	up
45891	hsa-miRPlus-E1285	1.284961847	3.321889255	up
46786	hsa-miRPlus-F1193	1.164771787	3.239755779	up
17354	hsa-miR-637	1.335324064	3.221411774	up
46563	hsa-miRPlus-F1202	1.30022367	3.173200525	up
42761	hsa-miR-675	1.218960357	3.163072795	up
145805	hsa-miR-765	1.572632897	3.130421605	up
42958	hsa-miR-628-3p	1.297814086	3.109545323	up
46630	hsa-miRPlus-F1064	1.131985734	2.970652306	up
42581	hsa-miR-513a-5p	1.226681539	2.911860284	up
145768	hsa-miR-665	1.181369241	2.801925761	up
27565	hsa-miR-423-5p	1.258361476	2.796250069	up
145938	hsa-miRPlus-E1151	1.619136229	2.750337221	up
42513	hsa-miR-300	1.235394649	2.642977256	up
42654	hsa-miR-483-5p	1.648160558	2.436035817	up
42531	hsa-miR-602	1.20758186	2.379348109	up
45614	hsa-miRPlus-F1155	1.15182669	2.314960093	up
42793	hsa-miRPlus-A1072	1.334835393	2.3062702	up
42467	hsa-miR-129-5p	1.155345627	2.296429439	up
46871	hsa-miRPlus-F1066	1.331027445	2.245377203	up
46561	hsa-miRPlus-E1168	0.955472363	2.235258446	up
46885	hsa-miRPlus-E1225	1.142350464	2.170793163	up
145846	hsa-let-7e	1.516590798	2.167017911	up
145801	hsa-miR-184	1.131662465	2.113451308	up
17953	hsa-miR-183^*^	1.123339786	2.102255691	up
42968	hsa-miR-202	1.442136753	2.014155741	up
146165	hsa-miR-1973	1.206740981	1.899462244	up
11024	hsa-miR-223	1.12320851	1.757335096	up
17327	hsa-miR-630	1.207656278	1.4871558	up
42490	hsa-miR-505^*^	1.038337575	0.864338657	down
42747	hsa-miR-93^*^	0.935485663	0.792346632	down
**10997**	**hsa-miR-19a**	**0.872578588**	**0.733001952**	**down**
46801	hsa-miR-106a	0.842840209	0.725109957	down
145745	hsa-miR-335^*^	1.014443604	0.684971442	down
17854	hsa-miR-106b^*^	0.945753297	0.682903417	down
**42588**	**hsa-miR-18a**	**0.944571773**	**0.651215282**	**down**
145878	hsa-miR-18b	0.964166256	0.627529305	down
**42650**	**hsa-miR-17**	**0.790386493**	**0.60498811**	**down**
17882	hsa-miR-20b^*^	0.826599873	0.59968123	down
**13178**	hsa-miR-18a^*^	**0.868819105**	**0.585715075**	**down**
10972	hsa-miR-181b	0.890432724	0.558983429	down
**145693**	**hsa-miR-92a**	**0.700276182**	**0.533072004**	**down**
46404	hsa-miR-1244	0.815394481	0.531463012	down
17718	hsa-miR-92b	0.687812065	0.524318954	down
46328	hsa-miR-1274b	1.01751262	0.522455132	down
42825	hsa-miR-888^*^	0.943134548	0.513072208	down
145915	hsa-miR-20b	0.73923086	0.512996216	down
14285	hsa-miR-487b	1.024734811	0.506362825	down
**19588**	hsa-miR-17^*^	**0.759996826**	**0.489222786**	**down**
27544	hsa-miR-363^*^	0.831476583	0.488030955	down
146025	hsa-miR-2277	0.790463344	0.467565743	down
46221	hsa-miR-519d	0.889689413	0.465041783	down
17377	hsa-miR-600	0.729438477	0.46279117	down
46737	hsa-miR-1265	1.00780685	0.46224256	down
46817	hsa-miRPlus-E1172	0.799968081	0.460279627	down
**42926**	hsa-miR-92a-1^*^	**0.587982974**	**0.430810607**	**down**
42872	hsa-miR-138-1^*^	0.797437183	0.355101118	down
46352	hsa-miRPlus-E1082	0.724440823	0.335770029	down
46649	hsa-miRPlus-E1012	0.656336839	0.301169524	down
46739	hsa-miR-1308	0.817749197	0.279223798	down
**42663**	hsa-miR-20a^*^	**0.490973444**	**0.273653825**	**down**
46537	hsa-miRPlus-E1038	0.767335506	0.270737134	down
10977	hsa-miR-183	0.67665388	0.2377643	down

miRNAs corresponding to miR-125a-5p and miR-17-92 cluster are in bold. FC, fold change relative to control untreated HL-60 cells (C); D2, HL-60 cells treated with DMSO for 2 days; D4, HL-60 cells treated with DMSO for 4 days. MiR^*^ nomenclature (an asterisk appended to the miRNA name) indicates a mature miRNA that originates from the same hairpin of the main miRNA, is complementary to the main miRNA and is less predominant than the opposite arm (miRNA without asterisk) of the hairpin.

Because miR-125a knockout mice have recently been shown to develop myeloproliferative disorders with a granulocyte hyperplasia [35], we further analyzed the role of this miRNA in neutrophil differentiation. On the other hand, our data showed that the miR-17-92 cluster was one of the most downregulated miRNAs during DMSO-induced HL-60 cell differentiation (Table 1 and Figure 2A). miR-17-92 cluster comprises six miRNAs: miR-17, miR-18a, miR-19a, miR-20a, miR-19b-1 and miR-92a-1, and is suggested to play an important role in hematologic malignancies, including AML [36, 37]. On these grounds, the role of this miRNA cluster in neutrophil differentiation was also further examined.

The data of the miRNA array analyses were validated by real-time quantitative polymerase chain reaction (RT-qPCR), analyzing the expression levels of miR-125a-5p, miR-16, miR-17, miR-20a, miR-22, miR-223, and miR-628-3p during HL-60 cell differentiation (Figure 2C). As shown in Figure 2C, the miRNA array and the RT-qPCR data indicated that miR-125a-5p, miR-16, miR-22, miR-223 and miR-628-3p were upregulated, whereas miR-17 and miR-20a were downregulated, using both experimental techniques (Figure 2C). Moreover, miR-223 was also upregulated in our miRNA screen (Figure 2A and Table 1), in agreement with previous results that linked this miRNA to the granulocytic lineage [12, 13, 38], further supporting the reliability of our experimental approach.

The annotation miRPlus in Table 1 and Figure 2A refers to Exiqon in-licensed human sequences not yet annotated in miRBase (last release version 21: June 2014) [39–41]. miRPlus miRNA candidates are predicted miRNA sequences derived from Exiqon’s database of proprietary material, database mining, and publications, and were included in the chip microarray as novel sequences. Hierarchical clustering analysis together with volcano plots showed 52 differentially expressed miRPlus miRNAs during DMSO-induced granulocytic HL-60 differentiation (Supplementary Figures 4 and 5); 25 of these new miRPlus sequences were upregulated, whereas 27 miRPlus sequences were downregulated.

### Functional role of upregulated miR-125a-5p in neutrophil differentiation of human leukemia HL-60 cells

**Figure 3:**
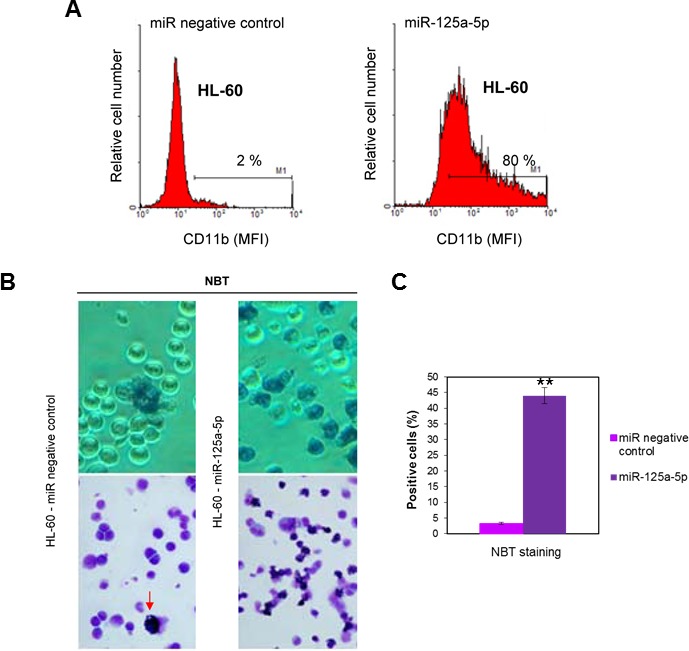
Ectopic expression of miR-125-a-5p promotes granulocytic cell differentiation of human myeloid leukemia HL-60 cell line. HL-60 cells were transfected with miR-125a-5p and miR negative control as shown in the Materials and Methods section, and cell differentiation towards the granulocytic lineage was analyzed following cell surface expression of CD11b by flow cytometry (**A**) or nitroblue tetrazolium (NBT) reduction (**B**) after 48-h incubation. Arrow shows a NBT-positive stained cell. Percentages of CD11b-positive cells are indicated in panel A. MFI, mean fluorescence intensity. Data shown are representative of three independent experiments. (**C**) Quantitative measurements of the percentages of NBT-positive cells in HL-60 cells transfected with miR-125a-5p and miR negative control. Data shown are means ± SD of three independent experiments. ^**^, *p* <0.01, with respect to miR negative control.

MicroRNA-125a-5p is a mammalian homolog of *C. elegans* lin-4, the first identified microRNA, which plays a critical role in development and differentiation [42]. In order to examine whether miR-125a-5p upregulation was involved in neutrophil differentiation of HL-60 cells, we analyzed how its enforced expression affected HL-60 cells. We found that transfection of undifferentiated HL-60 cells with pre-miR-125a-5p, designed to mimic endogenous miR-125a-5p, led to granulocytic differentiation, as assessed by a high increase in CD11b cell surface expression (Figure 3A) as well as in the number of nitroblue tetrazolium (NBT)-positive cells (Figure 3B and 3C), as compared to cells transfected with pre-miR negative control.

**Figure 4:**
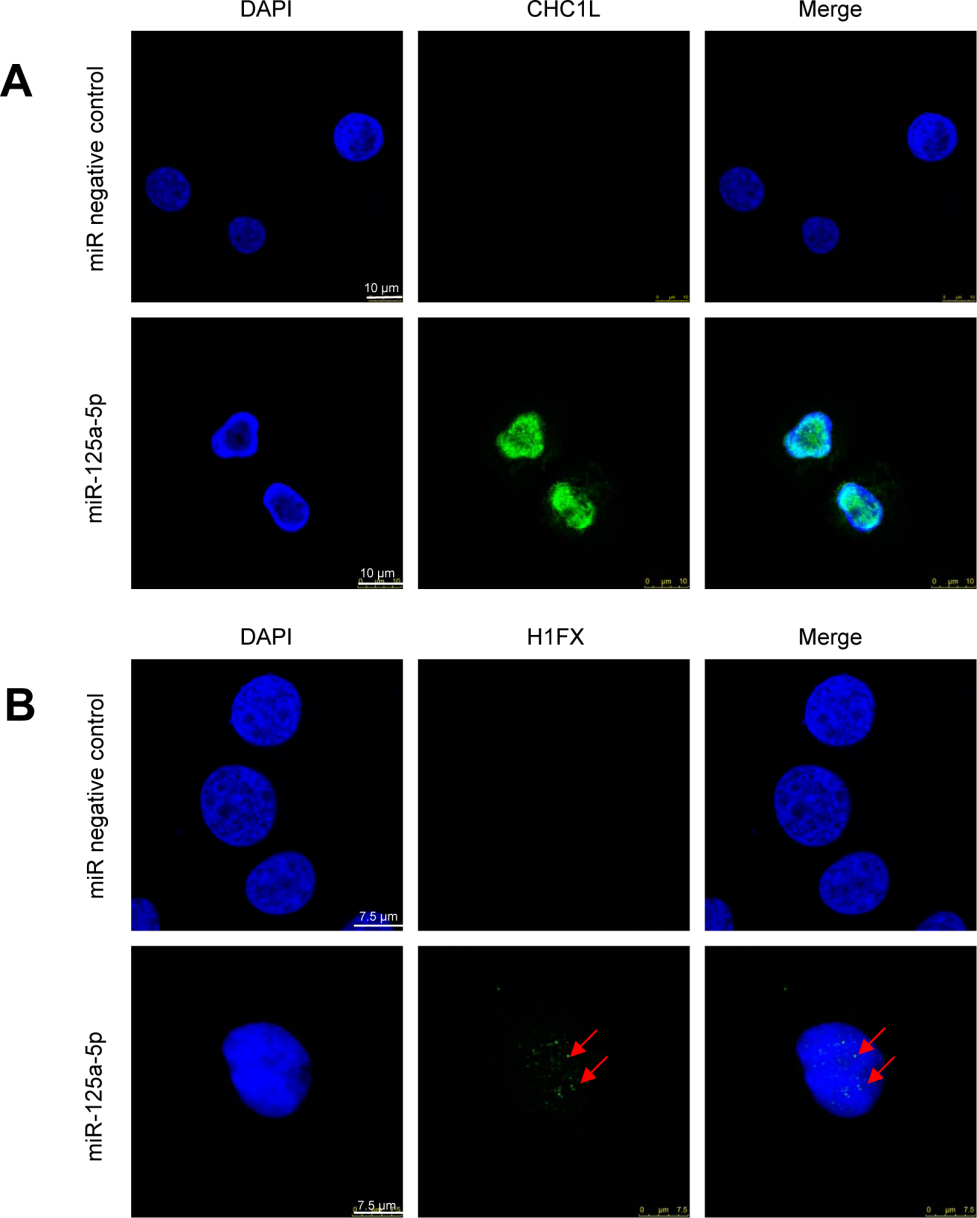
Confocal microscopy for the expression of CHCL1 and H1FX in HL-60 cells following miR-125a-5p ectopic expression. HL-60 cells were transfected with miR-125a-5p and miR negative control as shown in the Materials and Methods section, and expression of CHC1L and H1FX were analyzed by confocal microscopy. **(A)** CHCL1 expression was induced following miR-125a-5p transfection and co-localized with the nucleus stained in blue with DAPI. **(B)** H1FX was also upregulated following transient transfection of HL-60 cells with miR-125a-5p, as shown by arrows. DAPI stained in blue the nucleus. Images shown are representative of three independent experiments.

Chromatin remodeling is a major feature of neutrophil differentiation, leading to the acquisition of a rather peculiar nuclear morphology showing chromatin condensation [8, 43, 44]. Some genes related to chromatin remodeling, such as CHC1L (chromosome condensation 1-like) and histone H1FX (H1 histone family member X), have been found to be upregulated in previous mRNA microarray profiling studies during DMSO-induced granulocytic differentiation of HL-60 cells [29]. Here, we found that ectopic expression of miR-125a-5p induced the expression of the chromatin remodeling genes CHC1L and H1FX in the nuclei of HL-60 cells (Figure 4A and 4B), as assessed by confocal microscopy.

### Ectopic expression of miR-125a-5p induces granulocytic differentiation in human promyelocytic NB4 cells and primary CD34^+^-hematopoietic progenitor/stem cells (CD34^+^-HPCs)

**Figure 5:**
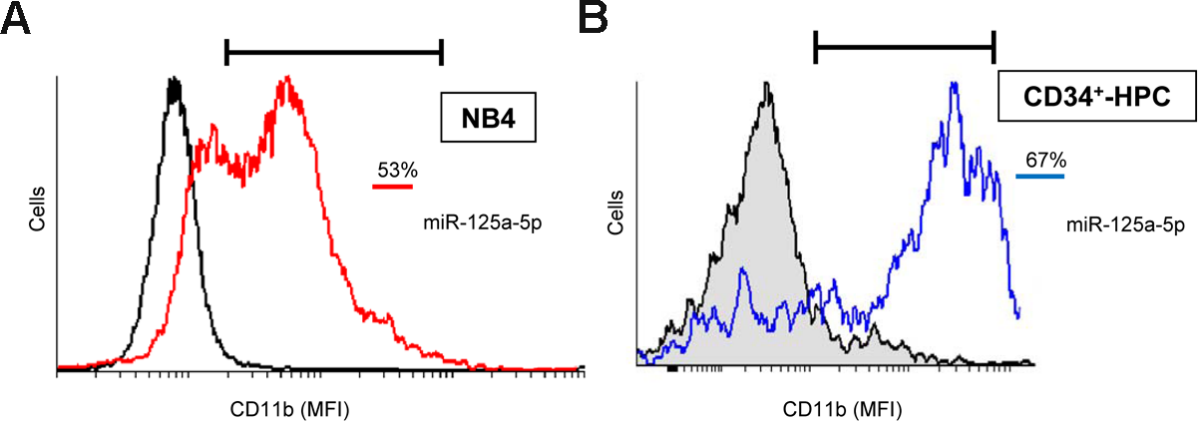
Ectopic expression of miR-125-a-5p promotes granulocytic cell differentiation of human promyelocytic NB4 cells and primary CD34^+^-HPCs. NB4 cells **(A)** and CD34^+^-HPCs **(B)** were transfected with miR-125a-5p (red line, NB4 cells; blue line, CD34^+^-HPCs) and miR negative control (black line) as shown in the Materials and Methods section, and cell differentiation towards the granulocytic lineage was analyzed following CD11b cell surface expression by flow cytometry. Percentages of CD11b-positive cells are indicated in **(A)** and **(B)** panels. MFI, mean fluorescence intensity. Data shown are representative of three independent experiments.

Enforced expression of miR-125a-5p also induced granulocytic differentiation in the human acute promyelocytic leukemia cell line NB4 (Figure 5A) as well as in primary normal CD34^+^-HPCs (Figure 5B), as assessed by a remarkable increase in the percentage of CD11b^+^-positive cells. The maturation of CD34^+^-HPCs towards neutrophilic lineage following miR-125a-5p ectopic expression was also confirmed by morphological analysis, in which the nucleus became polymorphic, multi-lobed and segmented (Supplementary Figure 6), similarly to the morphological changes shown in Figure 1D. These results extend the role of miR-125a-5p to different human acute myeloid leukemia cell lines as well as to normal human primary CD34^+^-HPCs to promote granulocytic differentiation, thus highlighting its major role in neutrophil differentiation of both normal human HPCs and AML cells.

### Functional role of downregulated miR-17-92 cluster in neutrophil differentiation of human leukemia HL-60 cells

**Figure 6:**
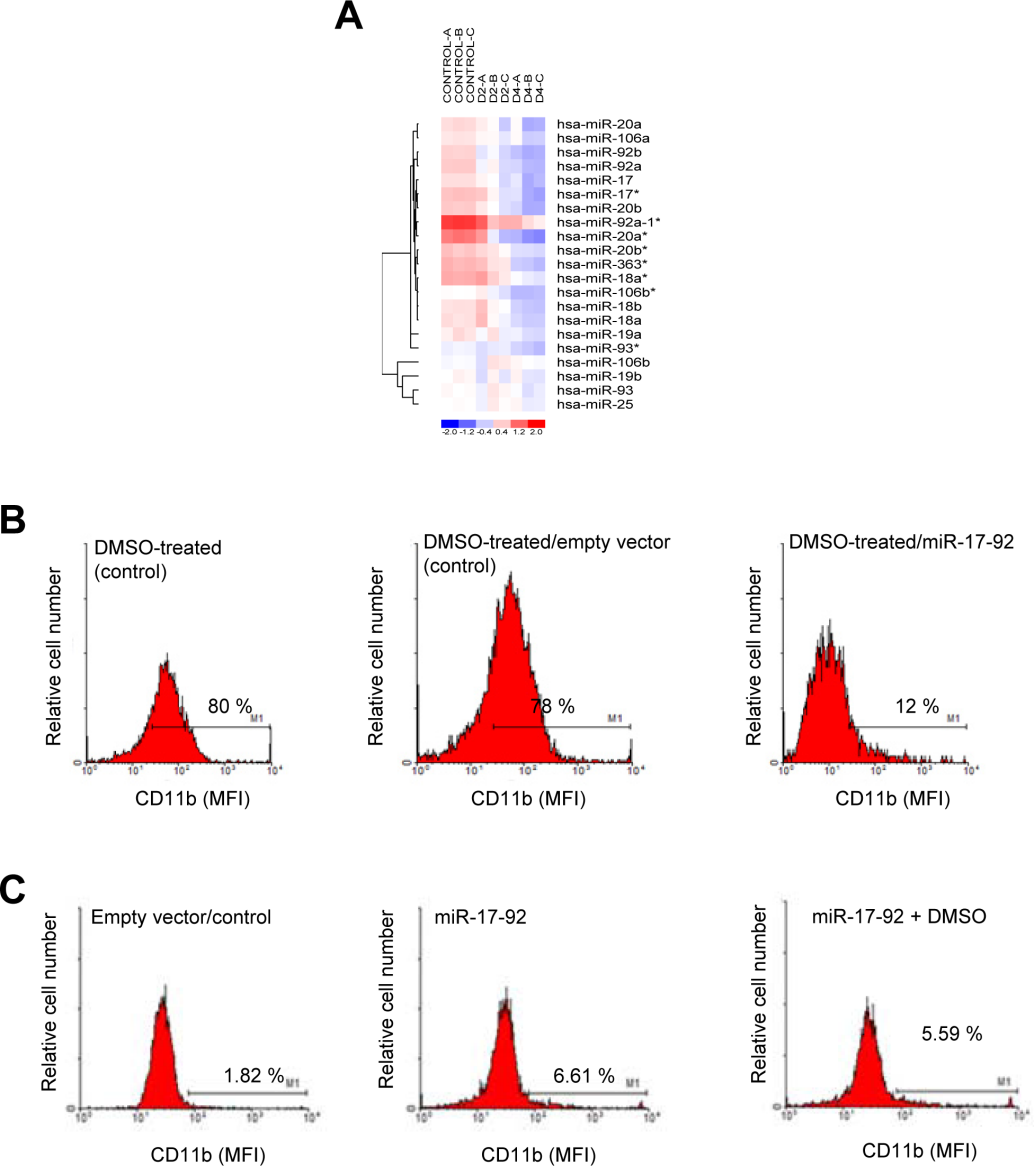
Effect of miR-17-92 cluster ectopic expression in HL-60 differentiation towards the granulocytic lineage. **(A)** Heat map and hierarchical clustering analysis of the distinct members of the miR-17-92 cluster and miRNA paralogs differentially expressed in untreated control versus DMSO-treated HL-60 cells for 2 (D2) and 4 (D4) days. Differential expression of miRNA patterns is shown by the intensity of red (upregulation) and blue (downregulation) colors. Rows: differentially expressed miRNAs; columns: untreated (Control) and DMSO-treated HL-60 cells for 2 (D2) and 4 (D4) days. Negative fold change (miRNAs with decreased expression) is represented in blue and positive fold change (miRNAs with increased expression) is represented in red. Three independent experiments were performed for each control and DMSO-treated cell population (Control, A-C; D2, A-C; D4, A-C). MiR^*^ nomenclature (an asterisk appended to the miRNA name) indicates a mature miRNA that originates from the same hairpin of the main miRNA, is complementary to the main miRNA and is less predominant than the opposite arm (miRNA without asterisk) of the hairpin. **(B)** Transient transfection with miR-17-92 blocks the differentiation of HL-60 cells induced by DMSO. HL-60 cells were electroporated with either empty vector (control, middle histogram) or miR-17-92 cluster (right histogram), and then (24 h later) cells were treated with 1.3% (v/v) DMSO for 4 days, and cell differentiation towards the granulocytic lineage was analyzed following cell surface expression of CD11b by flow cytometry. Untransfected HL-60 cells were also treated for 4 days with DMSO and run in parallel (DMSO-treated, control; left histogram). Untransfected and untreated HL-60 cells rendered less than 4% CD11b^+^ cells, similarly to controls shown in Figure 1A or Figure 3A. **(C)** Transient transfection of HL-60 cells with miR-17-92 does not promote granulocytic differentiation and prevents neutrophil maturation. HL-60 cells were transfected with empty vector (control) (left histogram) or miR-17-92 cluster (middle histogram), and, after 2 days in culture, CD11b cell surface expression was analyzed. HL-60 cells were also transfected with miR-17-92, and then immediately treated with DMSO for 2 days (right histogram). Cell differentiation was analyzed following CD11b cell surface expression by flow cytometry. Percentages of CD11b-positive cells are indicated in **(B)** and **(C)** panels. Data shown in **(B)** and **(C)** are representative experiments of three independent experiments.

As shown in Figure 2A, Figure 6A and Table 1, all the miRNAs of the miR-17-92 cluster were downregulated during HL-60 cell differentiation towards granulocytic lineage. Interestingly, not only the miR-17-92 cluster, but all the miRNAs of the two paralogs of the main cluster, named miR-106a-363 (comprising miR-106a, miR-18b, miR-20b, miR-19b-2, miR-19a-2 and miR-363) and miR-106b-25 (comprising miR-106b, miR-93 and miR-25) [37], were downregulated during the DMSO-induced differentiation of HL-60 cells (Figure 2A, Figure 6A, and Table 1). Figure 6A shows a miRNA expression heat map of the above three paralog clusters, namely miR-17-92, miR-106a-363 and miR-106b-25, showing downregulation of all the individual miRNAs. Interestingly, transfection of HL-60 cells with a miR-17-92 cluster overexpression plasmid (Addgene plasmid #21109) led to the inhibition of granulocytic differentiation in DMSO-treated HL-60 cells, as assessed by a decrease in the percentage of CD11b^+^-cells (Figure 6B). In addition, granulocytic differentiation of HL-60 cells was prevented when cells were transfected with miR-17-92 cluster and simultaneously treated with DMSO (Figure 6C). Taken together, these data suggest that downregulation of miR-17-92 cluster plays a major role in neutrophil differentiation of HL-60 cells, and further studies would be required to identify which specific miRs within the miR-17-92 cluster are directly involved in the granulocytic differentiation process.

### miR-125a-5p and miR-17-92 targetomes involving MAPK/ERK signaling and actin cytoskeleton

By using KEGG pathway analysis to examine the functional roles of predicted and validated miR-125a-5p and miR-17-92 target genes, we found that changes in the expression of these two miRNAs particularly affected genes involved in the MAPK/ERK signaling pathway (*p* values of 1.3 x 10^-10^ and 6.7 x 10^-9^ for miR-125a-5p and miR-17-92, respectively) (Supplementary Figures 7 and 8). MiR-17-92 target genes also include the regulation of actin cytoskeleton (*p* = 8.3 x 10^-6^) (Supplementary Figure 9). In this regard, a tight control of MAPK/ERK signaling has been shown to be essential in regulating proliferation and survival of CD34^+^-derived neutrophil progenitors, as well as the balance between proliferation and apoptosis during neutrophil differentiation [45]. The actin-based cytoskeleton is required for polymorphonuclear leukocyte motile functions including locomotion, shape change, phagocytosis, and adhesion [46]. KEGG analyses also showed that another major route affected by validated miR-125a-5p and miR-17-92 target genes (*p* values of 7.2 x 10^-9^ and 2.6 x 10^-10^ for miR-125a-5p and miR-17-92, respectively) was pathways in cancer (Supplementary Figures 10 and 11).

## DISCUSSION

Here we have identified that miR-125a-5p upregulation plays a critical role in the differentiation of human acute myeloid leukemia HL-60 and NB4 cells as well as of normal human CD34^+^-HPCs towards neutrophils or neutrophil-like cells. On these grounds, and because our results include distinct human acute myeloid leukemia cell lines and primary cultures of normal human CD34^+^-HPCs, we can conclude that upregulation of miR-125a-5p is crucial for neutrophil differentiation. This finding is supported by recent evidence showing the involvement of miR-125a as a positive regulator of granulopoiesis in mice [47]. *MiR125a* knockout mice has been recently shown to have decreased neutrophil numbers and reduced infiltration of neutrophils in the lung following endotoxin challenge, as a consequence of an impaired development of granulocyte precursors to mature neutrophils [47]. In addition, the results shown here indicate that downregulation of the miR-17-92 cluster is required for differentiation of human leukemia HL-60 cells towards mature neutrophil-like cells. These findings point out the important role of the above miRNAs, namely miR-125a-5p and miR-17-92 cluster in granulopoiesis. Previous miRNA profiling data conducted with human induced pluripotent stem cells [48], and with the distinct populations of bone marrow granulopoiesis [16], have described a number of miRNAs that are differentially regulated during neutrophil differentiation, but no further studies on their specific involvement and significance in driving neutrophil differentiation were carried out. Thus, very few individual miRNAs have been reported to be implicated in neutrophil development and function [18]. Only miR-223 has been suggested to be involved in granulocyte differentiation, likely through its effect on the transcriptional factor *Mefc2* [12], but the underlying mechanisms are still unknown. Despite there are still some contradictory data that cast some doubts on a direct role of miR-223 on granulopoiesis [12, 49–51], most of the current evidence shows a steadily increased expression of miR-223 during neutrophil maturation, with the highest expression in mature neutrophils [52], thus suggesting a role for miR-223 in typical features of mature neutrophils and in regulating recruitment of neutrophils into infected tissue sites [51]. In this regard, we have found an increase in miR-223 level along HL-60 cell differentiation to neutrophil-like cells, particularly after a 4-day DMSO treatment (Figure 2A and 2C, and Table 1), leading to fully differentiated neutrophil-like cells, and being consistent with a putative role in mature neutrophils.

The evidence shown here for the novel involvement of miR-125a-5p and miR-17-92 in the differentiation of human neutrophils is three-fold: a) miR-125a-5p is upregulated and miR-17-92 cluster is downregulated during neutrophil differentiation of leukemic HL-60 cells; b) ectopic expression of miR-125a-5p induces neutrophil differentiation in distinct human AML cell lines as well as in normal human CD34^+^-HPCs; c) ectopic expression of miR-17-92 cluster inhibits granulocytic differentiation in DMSO-treated HL-60 cells.

MiR-125 is a highly conserved miRNA family throughout different species from nematodes to humans, which has been shown to be involved in various cancer types [42]. This miRNA family consists of three homologs, miR-125a, miR-125b-1 and miR-125b-2, which are transcribed from different loci in the genome [53], and it is becoming clear that the miR-125 family members display diverse and even opposite functions in different cell contexts [54]. Thus, miR-125b has been reported to act as a tumor promoter in various cancers, being upregulated in several human leukemias, including acute and chronic myeloid leukemia [55–57]. In contrast, miR-125a has been shown to play a tumor suppressor function in a number of solid cancers [42, 58–62]. This miRNA seems to play a fundamental role in development and cell differentiation, showing an anti-proliferative activity as well as an ability to promote cell differentiation [42]. Functional characterization of the promoter of miR-125a-5p has linked its activity to the transcription factor NF-kB and the inflammatory response [62]. Thus, our results fit well with the role of miR-125a-5p in the differentiation towards neutrophils, an inflammatory cell type unable to proliferate. Furthermore, miR-125a is downregulated in AML patients [63], its ectopic expression leads to inhibition of cell proliferation and enhanced apoptosis in AML cells [64], and miR-125a knockout mice develop myeloid malignancies [35]. In this regard, our results reported here support a role of miR-125a-5p in the generation of cells unable to proliferate and ready to undergo spontaneous apoptosis like mature human neutrophils. Taken together, these data show the different and even opposing roles of miR-125a and miR-125b in cell differentiation. In humans, miR-125a is organized in a cluster with miR-99b and miR-let-7e on chromosome 19, and all of them were upregulated during DMSO-induced HL-60 cell differentiation towards granulocytes (Figure 2A and Table 1). This is not surprising as all three miRNAs are encoded close to each other (<1kb), suggesting that this cluster has only one promoter and is transcribed as one transcript [62, 65].

**Figure 7:**
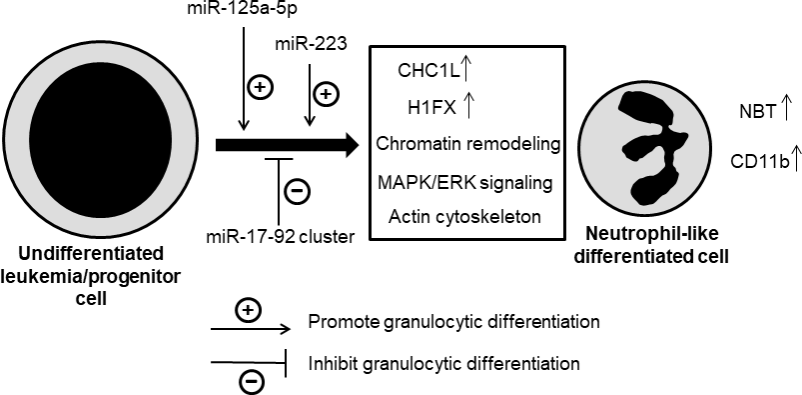
Schematic model of neutrophil differentiation by miRNAs. This is a schematic diagram designed to portray one plausible mechanism of how miRNAs influences at different stages of neutrophil differentiation based on the data reported here. Some miRNAs have positive (miR-125a-5p, miR-223) and others negative (miR-17-92) effects on the differentiation process, by affecting different signaling events that lead to differentiated neutrophil-like cells, showing an increased cell surface expression of CD11b and an enhanced NBT reduction activity. CHC1L, chromosome condensation 1-like; H1FX, H1 histone family member X. See text for details.

The polycistronic miRNA cluster miR-17-92, also called oncomir-1, has been shown to be frequently amplified and overexpressed in lymphoma, leukemia and several solid tumors, being considered one of the most potent oncogenic miRNAs [66–68]. MiR-17-92 has been suggested to play a major role in the development of mixed-lineage leukemia (MLL)-rearranged leukemias by inhibiting cell differentiation and apoptosis, whereas promoting cell proliferation, through the regulation of relevant target genes [36]. Some data have shown downregulation of miR-17-92 or of some of its members during mouse granulopoiesis [17], or during the different stages of human granulopoiesis [69]. Consistent with these data, we have found here that miR-17-92 cluster was downregulated during human leukemic HL-60 cell differentiation to mature neutrophil-like cells, which are non-dividing and apoptosis-committed cells. Furthermore, ectopic expression of miR-17-92 cluster had an inhibitory effect on the DMSO-induced differentiation towards granulocytes, thus suggesting that target genes of this miRNA cluster could act as promoters of neutrophil differentiation. Taken together, the results reported here provide the first evidence for the involvement of miR-125a-5p and miR-17-92 cluster in human neutrophil differentiation. Figure 7 depicts a schematic model for the involvement of the above miRNAs in neutrophil differentiation of human leukemia cells or normal human hematopoietic progenitor cells, leading to the morphological and functional changes characteristic of differentiated neutrophils or neutrophil-like cells. Given the large number of potential targets directly modulated by miR-125a-5p and miR-17-92, substantial effort is required to fully illustrate the modes of action of these miRNAs in neutrophil differentiation.

Our results, derived from a well-established and characterized human leukemia cell line (HL-60), widely used as a cell culture model for neutrophil differentiation and as a cell model to set up the basis of the differentiation therapy in acute promyelocytic leukemia [70, 71], together with additional results from primary human CD34^+^-HPCs and the additional acute promyelocytic cell line NB4, show compelling evidence for the identification of specific miRNA signatures involved in neutrophil maturation, and their dysregulation could lead to pathological conditions, such as cancer. Our data show how changes in specific miRNAs modulate neutrophil differentiation, and turn proliferating human leukemic myeloid cells in cells sharing features with mature neutrophils, which are end cells undergoing apoptosis, thus supporting miRNA-based therapy as a putative specific strategy for targeted cancer therapy and for differentiation therapy in leukemia.

## MATERIALS AND METHODS

### Cell culture

The human promyelocytic cell line HL-60 and NB4, were obtained from the American Type Culture Collection (ATCC) (Manassas, VA) and cultured in RPMI 1640 medium (Invitrogen, Carlsbad, CA) supplemented with 10% heat-inactivated fetal bovine serum, 2 mM L-glutamine, 100 U/ml penicillin and 100 μg/ml streptomycin at 37°C in 5% CO_2_. Exponentially growing cells (5-10 x 10^6^) were treated with 1.3% (v/v) DMSO (Sigma, St Louis, MO) for 2 and 4 days. Control undifferentiated cells were cultured without DMSO. Human CD34^+^-HPCs, derived from bone marrow (BM) (StemCell Technologies Inc., Vancouver, Canada) were cultured in HPMG™ hematopoietic growth medium (Lonza, Basel, Switzerland), supplemented with 50 ng/ml stem cell factor and 50 ng/ml FLT-3 ligand. Cell cultures consistently tested negative for mycoplasma contamination using the MycoProbe Mycoplasma Detection Kit (R&D Systems, Abingdon, UK).

### Transfection

Transient transfection was performed in 2 x10^6^ HL-60 and NB4 cells, as well as in 10^6^ CD34^+^-HPCs (StemCell Technologies Inc.), with 50 nM pre-miR miRNA precursor-miR-125a-5p (miR-125a-5p) or pre-miR miRNA precursor-negative control (miR negative control) (Thermo Fisher Scientific, Waltham, MA). Transient transfection was also performed in 2 x10^6^ HL-60 cells with 2 μg/ml pcDNA3.1/V5-His-TOPO-miR-17-92 (miR-17-92) plasmid [72] (provided by J. Mendell; Addgene plasmid #21109) or pcDNA3.1/V5 empty vector (control). The miR-17-92 cluster overexpression plasmid (Addgene plasmid #21109) leads to the expression of six mature miRNAs (miR-17, miR-18a, miR-19a, miR-20a, miR-19b and miR-92a) [73, 74]. Transfection was carried either by electroporation using a nucleofector kit (Amaxa Biosystems, Gaithersburg, MD) for CD34^+^-HPCs or lipofectamine 2000 (Invitrogen) for HL-60 and NB4 cells, following the manufacturer’s indications as well as previously reported electroporation conditions for CD34^+^-HPCs [75]. Transfection efficiency was 70-75%, as assessed using a plasmid carrying the enhanced version of green fluorescent protein (EGFP) as a reporter gene [75]. After 24 h transfection, HL-60 cells were induced to differentiate into granulocytes by treatment with 1.3% (v/v) DMSO for 4 days. In one set of experiments, HL-60 cells were transiently transfected with miR-17-92 cluster and simultaneously treated with 1.3% (v/v) DMSO for 2 (D2) days.

### Assessment of granulocytic differentiation

Granulocytic differentiation was evaluated by cell morphology with May-Grunwald-Giemsa-stained slides, NBT (Sigma) reduction activity (100-200 cells were counted), and by CD11b cell surface expression through flow cytometry, using an anti-CD11b Bear-I monoclonal mouse antibody [76] (provided by J.E. De Vries, Unicet, Lyon, France), as previously described [8, 21, 28, 77], or PE/Cy7 anti-human CD11b rat monoclonal antibody (1:100; Becton Dickinson, San Jose, CA). Flow cytometry analysis was performed using a FACSCalibur flow cytometer (Becton Dickinson, Franklin Lakes, NJ), and data were analyzed with FACSDiva software (Becton Dickinson).

### Apoptosis analysis by flow cytometry

Apoptosis was quantitated as the percentage of cells in the sub-G_0_/G_1_ region following cell-cycle analysis by flow cytometry, as previously described [78].

### Light microscopy

Cells in culture medium were centrifuged at 1500 rpm for 10 min, and the supernatant was removed. Cells were resuspended in cold phosphate-buffered saline (PBS), pH 7.4, and cytocentrifuged smears were prepared on glass slides at 500 rpm for 5 min (Cytospin 2, Shandom Southern Instruments, Inc., Sewickley, PA). The smears were fixed and stained using Wright-Giemsa and NBT staining. Smears were examined by light microscopy for NBT staining and nuclear morphology in 100-200 cells/smear, and data were expressed as percentages of total cells examined. For NBT and morphological analyses, the Mann-Whitney test was used and a *p* <0.05 was considered significant.

### Transmission electron microscopy

Untreated and treated HL-60 cells were rinsed briefly with PBS and then fixed for 1 h at room temperature with 2.5% glutaraldehyde buffered with PBS. After several rinses with PBS, cells were post-fixed for 1 h with 2% osmium tetra-oxide in H_2_O, rinsed in H_2_O and pelleted into 0.5% agar. Agar-embedded cells were then dehydrated in a graded ethanol series and embedded in Polybed 812 (Polysciences) epoxy resin. Thin sections of embedded cells were secondarily stained with uranyl acetate and lead citrate and examined in JEOL 100C electron microscope. Morphometric measurement of granules, mitochondria, cytoplasm and nuclei were performed on 20 random and consecutively photographed cells double stained with uranyl acetate and lead citrate.

### RNA extraction and miRNA microarray experiments

Total RNA was extracted from HL-60 cells using mirVana miRNA isolation kit (AM1560, Ambion/Applied Biosystem, Foster City, CA), according to the manufacturer’s instructions. RNA was quantified using a NanoDrop ND-1000 spectrophotometer (NanoDrop Technologies, Wilmington, USA), and checked for quality control and RNA Integrity Number by using Agilent 2100 Bioanalyzer (Agilent Technologies, Inc., Santa Clara, CA). Subsequently, RNA was run on a gel to visualize 18S and 28S rRNA subunits. Samples were labeled using the miRCURY™ Hy3/Hy5 Power labeling kit (Exiqon A/S, Vedbaek, Denmark), and hybridized on a hybridization station using Exiqon miRCURY™ LNA array v.14 (5^th^ generation - hsa, mmu and mo). The quantified signals were normalized using the locally weighted scatter plot smoothing (LOWESS) regression algorithm, which we have found to produce the best within-slide normalization to minimize the intensity-dependent difference between the dyes. Hierarchical clustering was performed to show distinguishable miRNA expression profiling among the samples.

### Validation of miRNA microarray results by RT-qPCR

To validate the miRNA microarray data, the expression of 7 miRNAs (miR-125a-5p, miR-16, miR-17, miR-20a, miR-22, miR-223 and miR-628-3p) was analyzed by RT-qPCR in the samples analyzed on the microarray. The relative expression of each one was determined by relative quantification as previously described [79]. The change in amplification was normalized to the expression of 3 reference miRNAs (miR-19b, miR-103 and miR-423-3p). Because the use of multiple control genes leads to more reliable and accurate normalization than the conventional use of a single gene [80], and the applicability of reference genes can vary between different studies [81], we identified stably expressed miRNAs that could serve as references for RT-qPCR by global mean normalization. MiR-19b, miR-103, miR-423-3p were selected as the miRNAs most resembling the behavior of the global mean, recognized as having the least variation after global mean normalization. These miRNAs were further evaluated by the use of NormFinder [82] and GeNorm [80] algorithms, used to determine the most stable reference genes from a set of candidate reference genes, and resulted the best reference miRNAs for normalization. Taken together, both algorithms pointed at miR-19b, miR-103 and miR-423-3p as the common best reference miRNAs for normalization, showing Normfinder and GeNorm stability values below the 0.50 cut-off for suitable reference genes [80, 82]. The fold change in expression of each target gene was calculated using the 2^-ΔΔCp^ method [79], where ΔΔCp=(Cp_target gene_-Cp_reference gene_)_treated_ – (Cp_target gene_-Cp_reference gene_)_untreated_. A 2^-ΔΔ^Cp value>1.5 or <0.67 was considered as a differentially expressed miRNA. RT-qPCR assays were performed in three independent experiments conducted in triplicate.

### Confocal microscopy

HL-60 cells were attached to slides by cytocentrifugation (500 rpm, 5 min). Cells for immunofluorescence were fixed for 20 min with 2% paraformaldehyde (methanol-free) in PBS and then permeabilized in 0.1% Tween-20 for 5 min. Anti-histone H1x (H1FX) rabbit polyclonal antibody (Abcam, Cambridge, UK), and anti-chromosome condensation 1-like (CHC1L) rabbit monoclonal antibody (Cell Signaling Technology, Danvers, MA) were used for immunofluorescence detection at a 1:50 or 1:100 dilution, and incubated overnight in a humid atmosphere at 4°C. Then, slides were washed and incubated with fluorescein isothiocyanate-conjugated goat anti-rabbit IgG (Cappel, Cochranville, PA) diluted 1:50 in PBS. DAPI was used at dilution of 1:1000 in PBS for nuclear staining. Images were taken with a Zeiss confocal laser scanning microscope (Oberkochen, Alemania) at x67 magnification.

### Molecular network analysis of differentially expressed miRNAs and miRNA target genes during differentiation of HL-60 cells

A set of miRNAs differentially expressed among untreated (control), and cells treated with DMSO for 2 (D2) and 4 (D4) days were identified by statistical analysis with one-way ANOVA (*p* <0.05). By using these miRNA as a discriminator, hierarchical clustering analysis was performed by using Cluster 3.0 (http://bonsai.hgc.jp/~mdehoon/software/cluster/software.htm) and TreeView 1.1.5r2 (https://sourceforge.net/projects/jtreeview/) software. Predicted and validated targets for the miRNAs identified in this work were obtained with the miRWalk 2.0 platform (http://zmf.umm.uni-heidelberg.de/apps/zmf/mirwalk2/) [83], along with the list of Kyoto Encyclopedia of Genes and Genomes (KEGG) pathways (https://www.kegg.jp/kegg/kegg1.html) [84–86] in which those targets were involved.

### Statistical analysis

Data are shown as means ± SD of the number of experiments indicated. Between-group statistical differences were assessed using Mann-Whitney, ANOVA or Student’s *t*-test. Statistical significance was defined as *p* < 0.05.

The differentially expressed miRNAs were identified by a fold change of >1.2. After normalization, significant differentially expressed miRNAs were identified through Volcano Plot filtering between the two experimental groups. Volcano plots are useful tool for visualizing differential expression patterns between two different conditions. The plots were constructed by plotting −log10 (p-value) on the y-axis and log2 (fold change) on the x-axis.

## SUPPLEMENTARY MATERIALS AND FIGURES


