# Supplementary material for: Genome-wide miRNA profiling and pivotal roles of miRs 125a-5p and 17-92 cluster in human neutrophil maturation and differentiation of acute myeloid leukemia cells
Source: Oncotarget. 2019 Sep 3;10(51):5313–31. doi: 10.18632/oncotarget.27123 (PMC6731105; doi:10.18632/oncotarget.27123)
Supplement: Supplementary file 1 [file oncotarget-10-5313-s001.pdf]

Genome-wide miRNA profiling and pivotal roles of miRs 125a-5p and 17-92 cluster in human neutrophil maturation and differentiation of acute myeloid leukemia cells

SUPPLEMENTARY MATERIALS

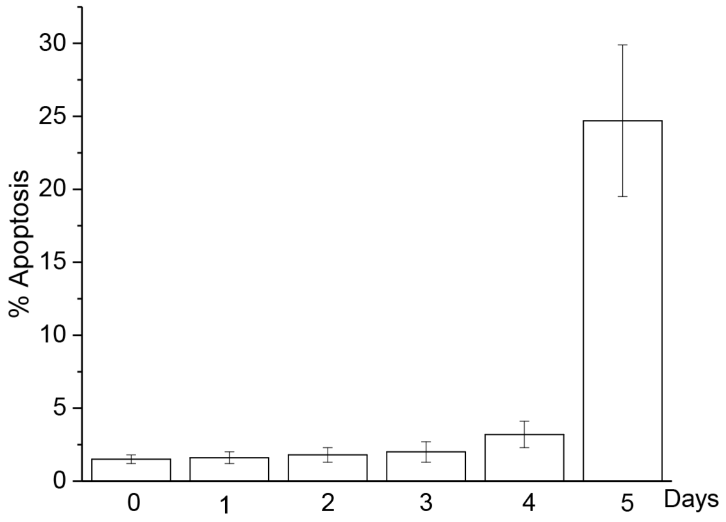

Supplementary Figure 1: Induction of apoptosis during DMSO-induced differentiation of HL-60 cells. DMSO (1.3 %, v/v) was added to the cell culture for the days indicated and apoptosis was determined by flow cytometry. Data shown are means ± SD of three independent experiments.

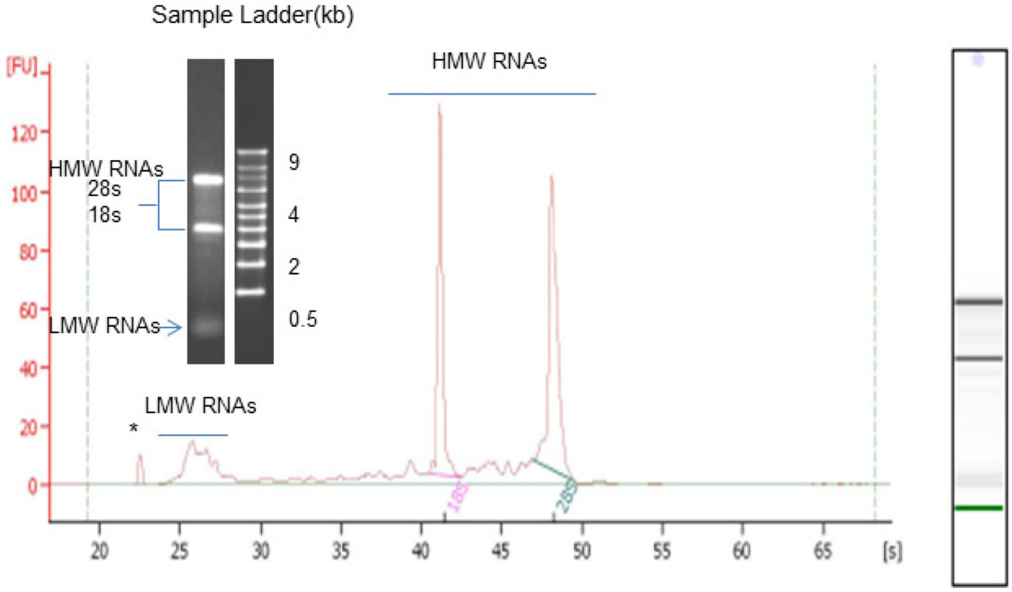

Supplementary Figure 2: RNA quality assessment. RNA electropherogram trace on 1.5% agarose gel to assess RNA quality, showing low (LMW) and high (HMW) molecular weight RNAs (*, loading marker).

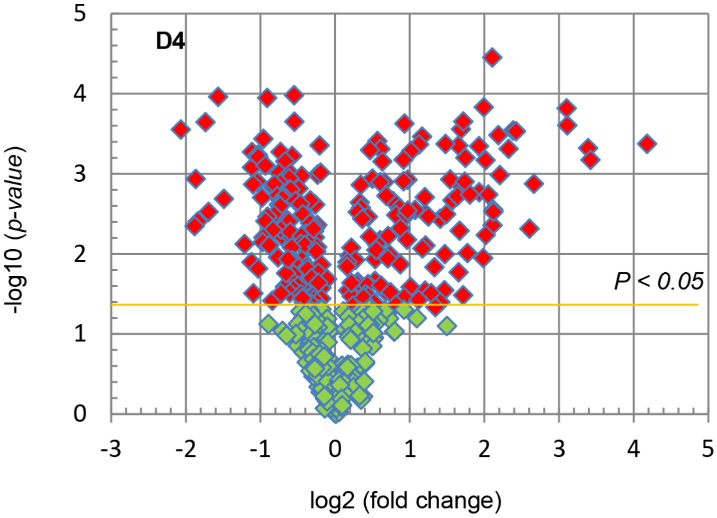

Supplementary Figure 3: Volcano plot analyses of the differentially expressed miRNAs (log 2 scale) versus the corresponding *p*-values (-log 10 scale), showing highly significant differentially expressed (up and downregulated) miRNAs after DMSO-induced differentiation of HL-60 cells for 4 days (D4).

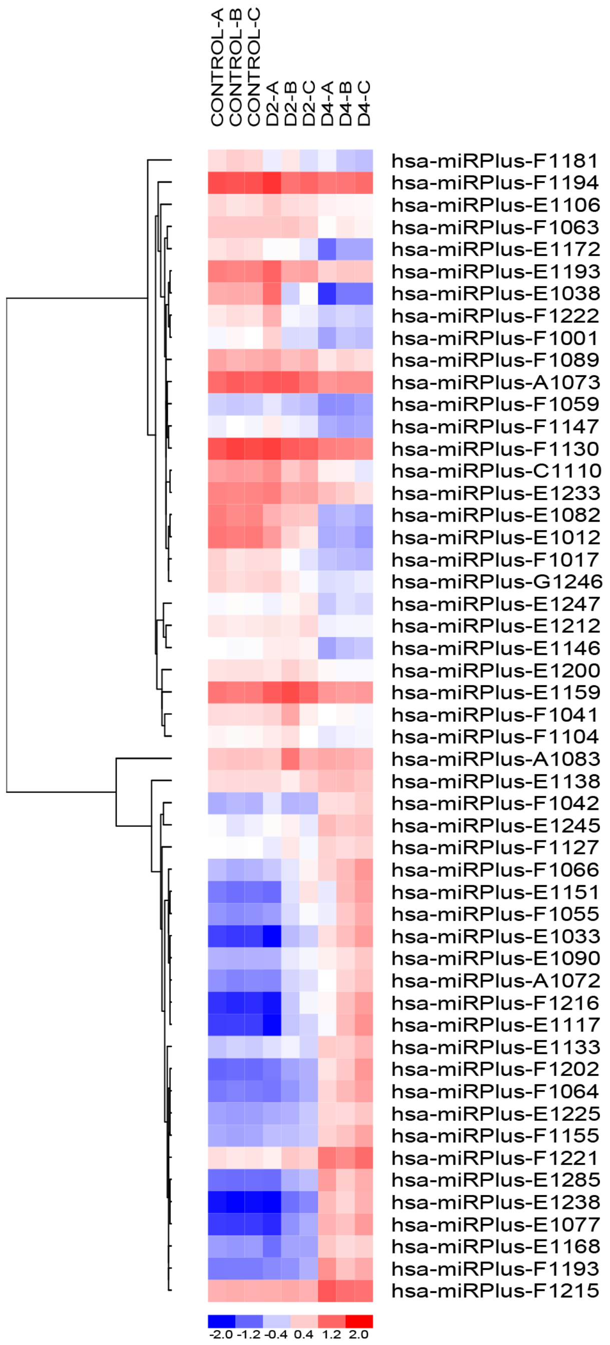

Supplementary Figure 4: Hierarchical clustering analysis of differentially expressed miRPlus microRNAs during DMSO-induced HL-60 cell differentiation. Control, untreated HL-60 cells; D2, 2-day DMSO-treated HL-60 cells; D4, 4-day DMSO-treated HL-60 cells. miRPlus profile expression in DMSO-treated cells was compared to undifferentiated control HL-60 cells. Data shown correspond to three different experiments (A, B, C).

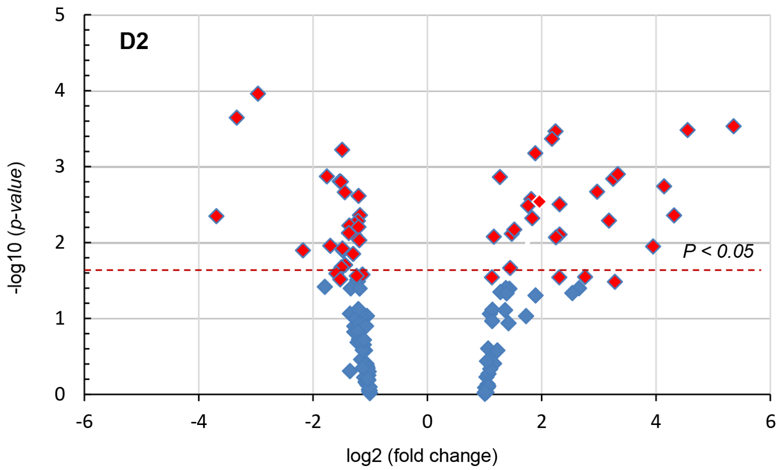

Supplementary Figure 5: Volcano plot analyses of the differentially expressed miRPlus microRNAs (log 2 scale) versus the corresponding *p*-values (-log 10 scale) were also carried out, showing highly significant differentially expressed (up and downregulated) miRPlus microRNAs after DMSO-induced differentiation of HL-60 cells for 2 days (D2).

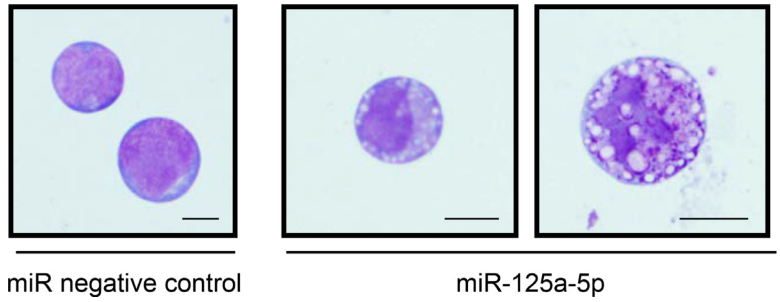

Supplementary Figure 6: Changes in cell morphology and nuclear shape following ectopic expression of miR-125a-5p in CD34^{+}-HPCs. Cell morphology and nuclear shape was assessed by Wright-Giemsa staining in CD34^{+}-HPCs transfected with miR-125a-5p and miR negative control as shown in the Materials and Methods section. *Bars*, 5 µm. Images are representative of three independent experiments.

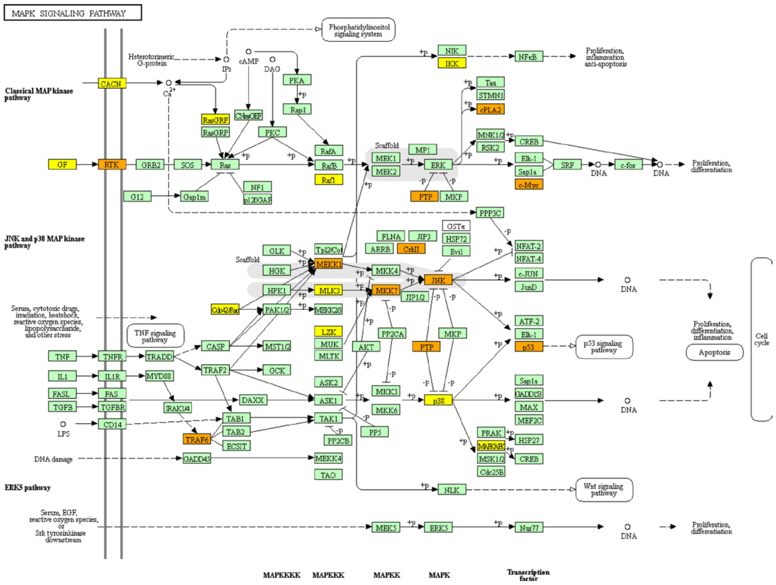

Supplementary Figure 7: Predicted (yellow) and validated (orange) miR-125a-5p target genes in the regulation of MAPK/ERK signaling KEGG pathway (https://www.kegg.jp/kegg/kegg1.html) identified with miRWalk 2.0 database (http://zmf.umm.uni-heidelberg.de/apps/zmf/mirwalk2/).

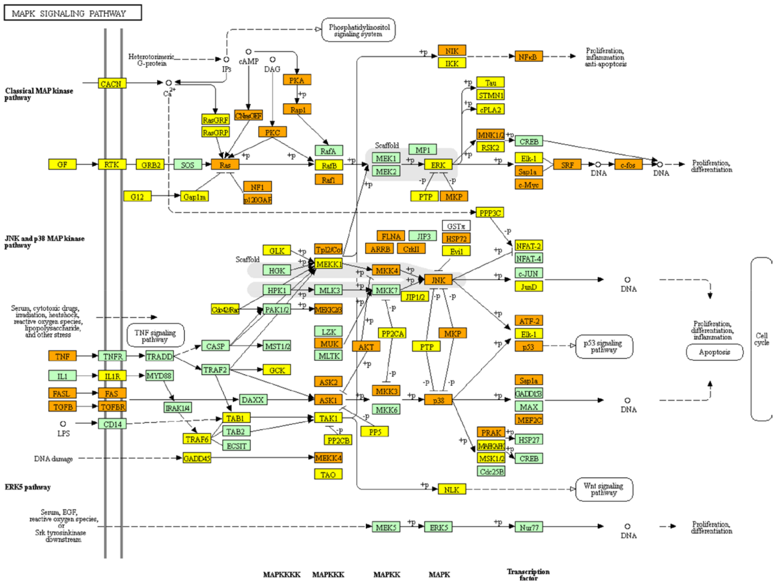

Supplementary Figure 8: Predicted (yellow) and validated (orange) miR-17-92 target genes in the regulation of MAPK/ERK signaling KEGG pathway (https://www.kegg.jp/kegg/kegg1.html) identified with miRWalk 2.0 database (http://zmf.umm.uni-heidelberg.de/apps/zmf/mirwalk2/).

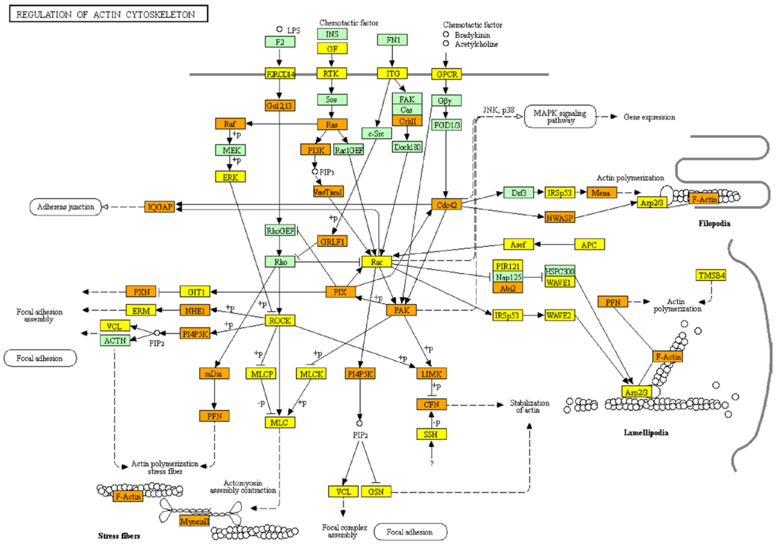

Supplementary Figure 9: Predicted (yellow) and validated (orange) miR-17-92 target genes in the regulation of actin cytoskeleton KEGG pathway (https://www.kegg.jp/kegg/kegg1.html) identified with miRWalk 2.0 database (http://zmf.umm.uni-heidelberg.de/apps/zmf/mirwalk2/).

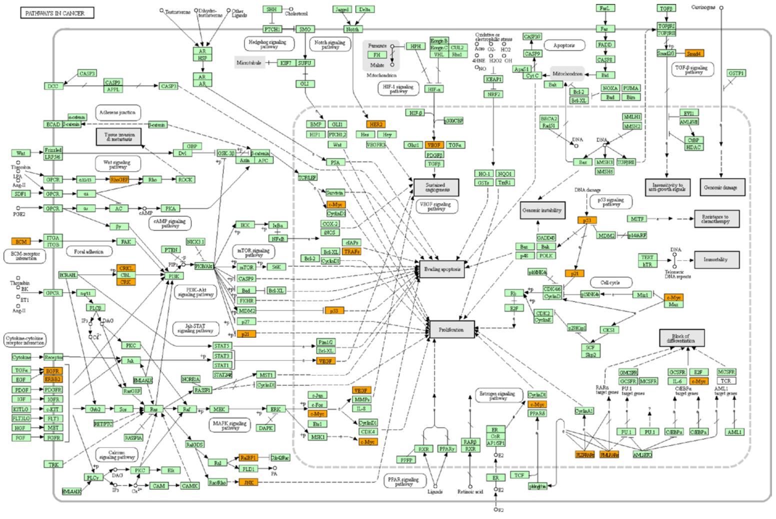

Supplementary Figure 10: Validated (orange) miR-125a-5p target genes in the KEGG pathways in cancer (https://www.kegg.jp/kegg/kegg1.html) identified with miRWalk 2.0 database (http://zmf.umm.uni-heidelberg.de/apps/zmf/mirwalk2/).

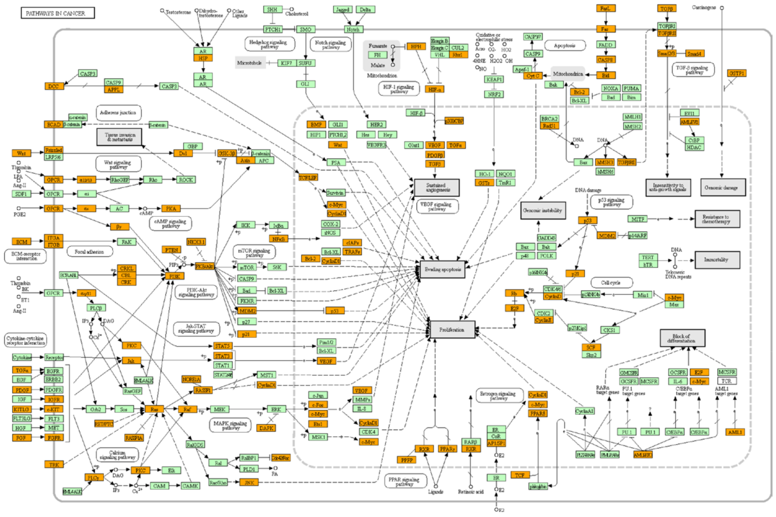

Supplementary Figure 11: Validated (orange) miR-17-92 target genes in the KEGG pathways in cancer (https://www.kegg.jp/kegg/kegg1.html) identified with miRWalk 2.0 database (http://zmf.umm.uni-heidelberg.de/apps/zmf/mirwalk2/).
